# Scientific Evidence of the Beneficial Effects of Tomato Products on Cardiovascular Disease and Platelet Aggregation

**DOI:** 10.3389/fnut.2022.849841

**Published:** 2022-03-15

**Authors:** Montaña Cámara, Virginia Fernández-Ruiz, María-Cortes Sánchez-Mata, Rosa M. Cámara, Laura Domínguez, Howard D. Sesso

**Affiliations:** ^1^Department of Nutrition and Food Science, Faculty of Pharmacy, Complutense University of Madrid, Madrid, Spain; ^2^Harvard Medical School, Brigham and Women's Hospital, Boston, MA, United States

**Keywords:** cardiovascular disease, platelet, antiplatelet, tomato, lycopene, health claims

## Abstract

Cardiovascular disease (CVD) includes a group of disorders of the heart and blood vessels that includes numerous problems, many of which are related to the process called atherosclerosis. The present work is aimed to analyze the most relevant studies examining the potentially beneficial effects of tomato products on both CVD prevention and antiplatelet aggregation as well as an European Food Safety Authority health claims evaluation on tomato and tomato products. To date, only one health claim has been approved for a concentrated extract of tomato soluble in water (WSTC) marketed under the patented name of Fruitflow® with two forms of presentation: WSTC I and II, with the following claim “helping to maintain normal platelet aggregation, which contributes to healthy blood flow.” Other studies also demonstrate similar beneficial effects for fresh tomatoes, tomato products and tomato pomace extracts.

## Introduction

The World Health Organization (WHO) defines cardiovascular disease (CVD) as disorders of the heart and blood vessels originated from a chronic inflammatory vascular process that affects the wall of medium-sized arteries and ends up producing endothelial dysfunction and atherosclerosis. An important intermediate consequence of CVD is endothelial dysfunction, an alteration characterized by the functional loss of the vascular system that precedes the morphological changes characteristic of atherogenesis (1). Longer-term clinical trials examining clinical cardiology outcomes often define major cardiovascular events to include non-fatal myocardial infarction, non-fatal stroke, and CVD death.

According to the latest study published in 2020 by the American College of Cardiology Foundation on the global burden of CVD (Global Burden Disease, GBD) and its risk factors, CVD is the leading cause of death and disability in the world. Despite improvements in our knowledge of the primary prevention of CVD, progress remains muted–particularly among second- and third-world countries with limited access to preventive services. As a result, in the last 30 years (1990–2019), there has been a marked and worrying increase in the number of cases (48.2%), deaths (35.7%) and disability (48.5%) due to CVD (2).

Although CVD is multifactorial, one major risk factor is high plasma concentrations of low-density lipoprotein (LDL). Oxidative stress is another important risk factor because of the imbalance between the body's oxidation-antioxidant processes. In this situation, the endogenous defense system is overcome by the formation of reactive oxygen species (ROS) that interact with different biomolecules (carbohydrates, lipids, proteins, amino acids, and nucleic acids) and cause cellular damage. Platelets also play a relevant role in these conditions, since it has been shown that platelet hyperaggregability is associated with an increased risk of coronary heart disease (1, 3).

For this reason, the role of diet is crucial in the development and prevention of CVD. Recommendations from national and international guidelines are to follow a diet low in saturated fats and rich in bioactive compounds such as antioxidants; to achieve this goal the inclusion of fresh fruit and vegetables, as tomato fruit and tomato products, is a valuable strategy (4).

## Health Claims Related to Tomatoes and Its Bioactives

Health claims in the labeling, presentation and/or advertising of food products are regulated in Europe by Regulations (EC) No. 1924/2006 of the European Parliament and of the Council; and Commission Regulation (EU) No. 432/2012. According to this legislation, scientific evidence on the role of a food, nutrient and/or compound in a nutritional or physiological function is not sufficient to justify the claim of beneficial effects. The substance must be present in the final product in sufficient quantities and, in addition, the amount of food that is necessary to consume to obtain the nutritional or physiological effect must be reasonable and easily achievable within the context of a balanced diet (5, 6). EFSA establishes that to consider a food or one of its constituents as subject of any health claim, it must demonstrate a beneficial effect. In this case, it would correspond to the maintenance and/or improvement of cardiovascular function, or the reduction of a risk factor for the development of this disease (7).

For lycopene, the main bioactive compound in tomato fruits, EFSA has published five scientific opinions on the approval of claims in relation to cardiovascular health and its risk factors, two of which refer to a water soluble tomato concentrate (WSTC), and three scientific opinions on lycopene (8, 9).

The first opinion was published in 2009 and evaluated the possible cause-effect relationship between the intake of a tomato extract preparation containing lycopene and whey proteins (lycopene-whey complex), and the reduction of the risk of atherosclerotic plaque formation by preventing oxidation of plasma lipoproteins. This beneficial effect was attributed to lycopene, which was sufficiently characterized. Eighty publications were evaluated, including six intervention studies, 22 observational studies, and eight reviews. The Panel reported several limitations in the design of these studies (sample size, duration, dose, absence of control groups, etc.), so a cause-effect relationship between lycopene intake and effect could not be established (7).

The second opinion was published in 2011 and evaluated the possible cause-effect relationship between the intake of lycopene and the protection of DNA, proteins, and lipids against oxidative damage due to its antioxidant capacity, as well as its contribution to normal cardiac function. According to the NDA Panel, lycopene was sufficiently characterized, and its main dietary sources corresponded to tomato and its derivatives. While a large number of scientific studies were provided in support of the health claim, many studies did not include original data since they were narrative reviews and consensual opinions. Other studies included results not related to the alleged effects or were focused on other bioactives such as carotenoids or antioxidant vitamins, alone or in combination with lycopene. In addition, none of the studies demonstrated a significant effect of lycopene on reliable markers of oxidative damage vs. controls. Finally, there was inconsistency in the provided studies that showed either no, negative, or positive associations between lycopene (either intake and/or plasma concentrations) with the risk of CVD. For all of these reasons, EFSA concluded that a cause-effect relationship could not be established between the intake of lycopene and its proposed beneficial effects (5).

The third opinion was published in 2015 and evaluated the possible cause-effect relationship between a lycopene preparation (named L-tug) obtained from an oleoresin extract from ripe tomato fruits mixed with other ingredients, and the reduction of the concentrations of plasma LDL-cholesterol. As it is a novel formulation, very limited information was provided from eight unpublished human intervention studies. The NDA Panel reported several limitations in the design of these studies, such as the absence of randomization and the lack of consideration of certain important methodological aspects, so that a cause-effect relationship could not be established between the intake of the lycopene preparation L-tug and the alleged effect (10).

Consequently, and according to the EFSA scientific opinions described above and published in 2009, 2011, and 2015, there has been insufficient scientific evidence to corroborate the alleged effects of lycopene on cardiovascular health and its risk factors for translation to published health claims on CVD and related outcomes to date.

**Figure 1:**
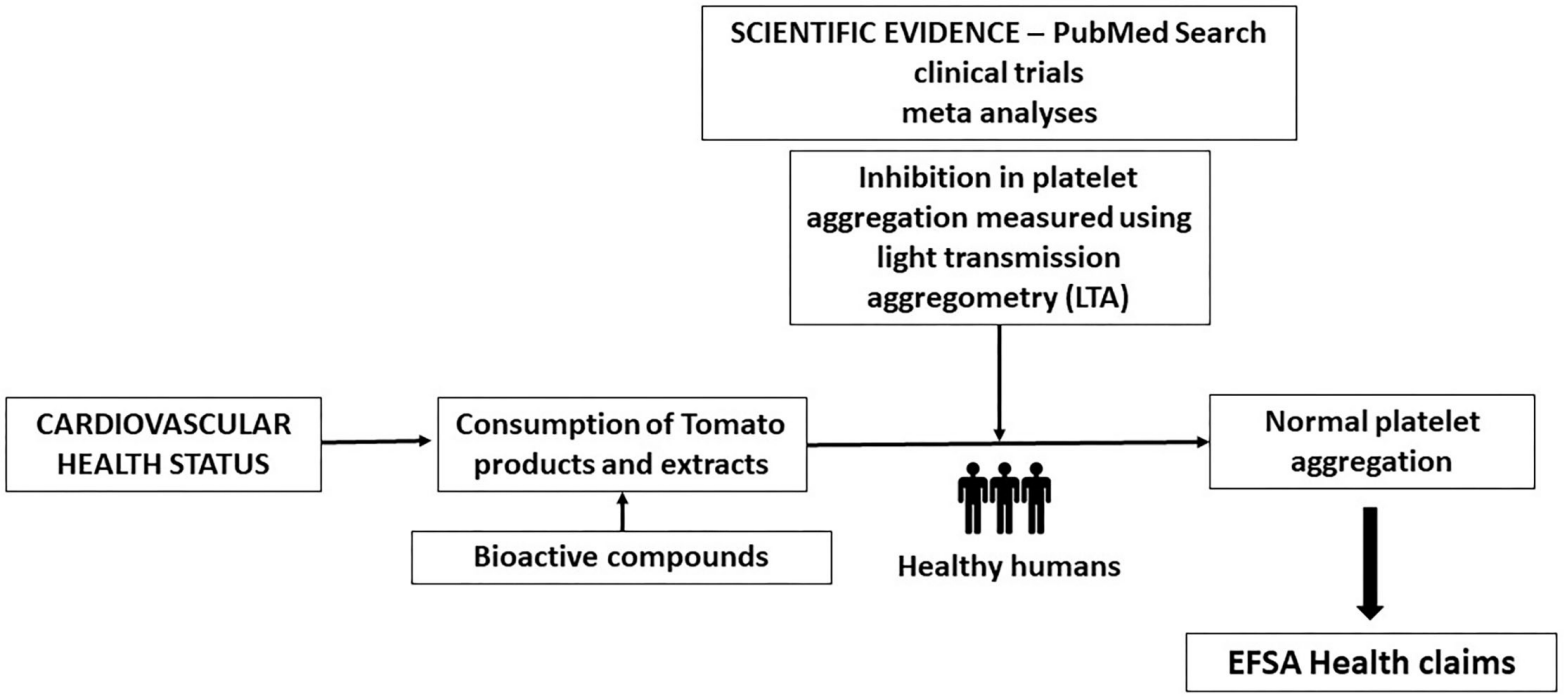
Scheme of the scientific evidence on the relationship between tomato and lycopene consumption, and antiplatelet aggregation effect.

This review is therefore aimed to review and analyse the scientific evidence for tomato products on CVD prevention and its anti-platelet effects in the context of its potential health claims according to EFSA requirements (Figure 1):

- Provide information proving that the consumption of the food/constituent (tomato-based product) reduces (or beneficially affects) platelet aggregation.
- Human clinical trials performance in subjects with platelet activation during sustained exposure to the food/constituent (at least 4 weeks).
- Use of valid markers: the percentage of inhibition in platelet aggregation should be measured using light transmission aggregometry (LTA) according to well-accepted and standardized protocols.

## Methodology: Literature Search

An extensive literature search in the following platforms and databases was performed in the years ranging from 2011 to 2021:

- Official digital platforms of the organizations involved in European food legislation: European Commission (European Commission, CE); European Parliament and Council of the European Union (European Parliament and Council of the European Union) and European Food Safety Authority (European Food Safety Authority, EFSA).
- PubMed database [https://pubmed.ncbi.nlm.nih.gov] following EFSA's approach was conducted. In a first search, the selected keywords were “tomato” and “cardiovascular disease,” in the second search the keywords used were “tomato” as well as the WHO disorder classification: “coronary heart disease,” “cerebrovascular disease,” “peripheral arterial disease,” “rheumatic heart disease,” “congenital heart disease” and “deep vein thrombosis.” In the third search, the following keywords were used: “tomato,” “platelet” and “antiplatelet.”

## EFSA Tomato and Lycopene Health Claims Requirements and Status

In order to demonstrate the beneficial effects of tomato product on lipid oxidative damage (lipid peroxidation), EFSA requires *in vivo* studies performance. In addition, the measurement of the following markers is required: changes in F2-isoprostanes in 24-h urine samples; measurement of oxidized LDL particles in the blood using immunological methods (antibodies) with appropriate specificity and the quantification of phosphatidylcholine hydroperoxides (PCOOH), measured in the blood or tissue by High-Performance Liquid Chromatography (HPLC) (1, 5).

According to the official database of the European Commission “EU Register on nutrition and health claims,” EFSA has evaluated 30 requests for approval of health claims referred to tomatoes and/or lycopene, both as a specific and individual compound, component of a food or constituent of a mixture in a commercial product. The great majority of the requests (24 of 30 requests) correspond to article 13.1 “general function,” while most other requests refer to article 13.5 “new function” (four of 30 requests) based on the latest scientific evidence and/or under the data protection, and article 14.1.a (two of 30 requests) relating to reducing the risk of illness.

Of all the submitted applications, 15 of 30 requests refer to lycopene as a component of tomato extracts or some derivatives (juice, pulp, and sauces) and 11 of 30 requests, relate lycopene with cardiovascular health and/or its risk factors (oxidative damage, high plasma cholesterol concentrations, and formation of atherosclerotic plaques) (11).

Of the 30 applications submitted, 29 obtained an unfavorable scientific opinion and only one health claim was approved, under article 13.5. The approved health claim is for a concentrated extract of tomato soluble in water (WSTC). This extract is marketed under the patented name of Fruitflow® (FF) with two forms of presentation: WSTC I and II. The effect claimed and approved by EFSA consisted of “helping to maintain normal platelet aggregation, which contributes to healthy blood flow” (11). Scientific evidence for Fruitflow ® included 15 studies (8 in humans and 7 in animals) which demonstrated that 37 compounds present in both forms of concentrated tomato extract (mainly nucleoside derivatives, conjugated phenolic compounds, and flavonoid derivatives) could significantly inhibit platelet aggregation (1, 12). Importantly, the 37 bioactive compounds identified in WSTC are naturally found in the starting product (that is, in tomato) and the concentration of soluble solids contained in WSTC roughly corresponds to the existing content in 2.5 tomatoes (13). To verify this information, O'Kennedy et al. (12) quantified the content of the bioactive compounds responsible for the alleged effect and that are present both in Fruitflow® and in tomato extracts and other derivatives (juice, tomato paste). The concentration of nucleoside derivatives and conjugated phenolic compounds was found to be higher in tomato (8,095.6 μg/g and 410.3 μg/g, respectively) compared to the patented WSTC extract (7,874.6 μg/g and 389.2 μg/g). The content corresponding to flavonoid derivatives was slightly lower in tomato (1,802.3 μg/g compared to 2,141.3 μg/g found in WSTC). According to O'Kennedy et al., other products, such as tomato juice and paste, showed slightly lower contents than those quantified in tomato extracts and Fruitflow®.

## Scientific Evidence on The Relationship Between Consumption of Tomatoes and Its Bioactive Derivatives and CVD Prevention

In our first literature search, with the selected keywords “tomato” and “cardiovascular disease,” we identified a total of 330 studies (167 and 92 in the last 10 and 5 years, respectively). These included four meta-analyses as the studies with the highest level of scientific evidence (14–17) which refer to 17 and 10 clinical trials in the last 10 and 5 years, respectively, as well as 6 systematic reviews (14–19).

The meta-analyses and systematic review carried out by Cheng et al. (14, 15), include 32 studies in humans to examine the possible relationship between plasma levels of lycopene and the risk of CVD. These studies also confirmed a significant reduction in systolic blood pressure (up to 5.66 mmHg) after supplementation with lycopene. Furthermore, Cheng et al. (15) reported that individuals with the highest plasma lycopene concentrations had a 26 and 37% lower risk of suffering a myocardial infarction and CVD mortality, respectively.

Li et al. (16) performed an umbrella review to collectively and systematically integrate individual study data, evaluate information from multiple meta-analyses on all health outcomes, and provide a wide view of the evidence landscape on tomatoes and lycopene. One hundred and seventy four articles were initially found, but only 17 articles with 20 health outcomes were identified based upon stringent eligibility criteria. Results showed that tomato intake is inversely associated with coronary heart disease mortality and CVD. The authors concluded that tomato or lycopene intake was generally safe and beneficial for multiple health outcomes in humans, but the quality of the evidence was not high.

Finally, Rattanavipanon et al. (17) conducted a systematic review and network meta-analysis on the effects of tomato intake, lycopene intake, and related food products on blood pressure in eight studies (*N* = 617 individuals), including seven trials (*N* = 501 individuals) in the analysis of systolic and diastolic blood pressure outcomes, respectively. Tomato products included standardized tomato extracts, a tomato- containing product without lycopene, and synthetic lycopene. Results showed that a standardized tomato extract significantly decreased systolic blood pressure compared to placebo, whereas the effect on diastolic blood pressure was not significant. In addition, other tomato products did not show consistent and significant effects on both systolic and diastolic blood pressure.

We then performed a second literature search using keywords like “tomato” as well as the WHO disorder classification (Table 1) results in: “coronary heart disease” (44 studies), “cerebrovascular disease” (28 studies), “peripheral arterial disease” (two studies) and “deep vein thrombosis” (one study).

**Table 1:** Summary of the studies found in PubMed by using the key words: “tomato” and the different types of CVD, according to WHO classification.

**Keywords**	**Total** **number** **of** **studies**	**Type of** **study**	**Studies** **in the** **last 10** **years**	**Studies in** **the last 5** **years**	**Relevant** **studies**
Tomato and coronary heart disease	44	1 meta- analysis; 7 clinical trials; 12 reviews	Total: 16; 1 meta- analysis; 2 clinical trials	Total: 9; 1 meta- analysis; 2 clinical trials	Meta- analysis: (16); Clinical trials: (18, 20)
Tomato and cerebrovascular disease	28	1 meta- analysis; 1 clinical trials	Total: 16; 1 Meta- analysis; 1 clinical trials	Total: 6; 1 Meta- analysis; 1 clinical trials	Meta- analysis: (16); Clinical trials: (21).
Tomato and peripheral arterial disease	2	Research articles	Total: 2	Total: 1	–
Tomato and deep vein thrombosis	1	Research article	Total: 1	–	–

## Scientific Evidence on the Relationship Between Tomato and Lycopene Consumption, and Antiplatelet Aggregation Effect

The literature search conducted in this review using the official Pubmed database and the keywords: “tomato,” “platelet” and “antiplatelet” resulted in a total of 29 studies. Most of them were reviews and included only seven original clinical trials, (12, 20, 22–26) (Table 2).

**Table 2:** Characteristics of the clinical trials found in PubMed by using the keywords: “tomato,” “platelet,” “antiplatelet,” and its compliance with EFSA requirements for anti-platelet effect health claim.

**References**	**Type of tomato** **product**	**Study** **performed** **on healthy** **subjects**	**Duration of** **the study: 4** **weeks of** **exposure** **(28 days)**	**Analytical** **EFSA** **valid** **markers**.
(20)	Tomato extract	NO (high-risk hypertensive patients)	YES	NO
(22)	Tomato juice	NO	–	–
(23)	Tomato extract (2 different extract- supplemented treatment drinks)	YES	NO	YES
(24)	Tomato extract (2 treatment supplement drinks using orange juice as a vehicle)	YES	NO	YES
(12)	Water-soluble tomato extract, Fruitflow (FF)	YES	YES	YES
(25)	Tomato pomace (byproduct of tomato pomace) extract (1 g, 2.5 g or placebo)	YES (male, *n* = 99)	NO	YES
(26)	Tomato extract Fruitflow®	NO	NO	NO

The information included in the most recent reviews (1, 27–31), and the clinical trials are discussed below.

Regarding the evidence on the beneficial effects of tomato products consumption in healthy people, Sesso et al. (32) conducted a prospective cohort study examining the intake of tomato and tomato juice in 27,267 healthy women free of baseline CVD or cancer. Results indicated that those subjects with a weekly consumption equal to or >10 servings of tomato and tomato products had lower concentrations of total triglycerides, low-density lipoprotein (LDL) cholesterol, and glycated hemoglobin (HbA1c), biological markers of cardiovascular risk. Li et al. (33) conducted a study with 25 young women (20–30 years old) who consumed 280 ml of tomato juice per day for 2 months. A significant reduction in plasma cholesterol levels was observed in all women, as well as an increase in the concentration of adiponectin, a hormone with anti-inflammatory and antiatherogenic properties that modulates the synthesis of nitric oxide (essential in endothelial function) and the proliferation of smooth muscle cells (present in blood vessels, among others). In addition, adiponectin protects against LDL oxidation. An *in vivo* study carried out by Hsu et al. (34) showed that the consumption of tomato paste for 8 weeks contributed to the reduction of plasma concentrations of total and LDL cholesterol, as well as an increase in plasma high-density lipoproteins (HDL) cholesterol and the activity of antioxidant enzymes such as catalase, superoxide dismutase, and glutathione peroxidase.

Burton-Freeman et al. (35) suggested that the consumption of tomato products could also attenuate the oxidation of LDL. This beneficial effect was observed in 25 individuals after eating foods with a high fat content. Postprandial oxidative stress was mitigated by the consumption of these tomato derivatives. Similarly, a decrease in lipid peroxidation, as well as an improvement in the general antioxidant status, was observed by García-Alonso et al. (36) in 18 healthy women who consumed tomato juice for 2 weeks. Xaplanteris et al. (37) also reported this effect in 19 individuals who consumed 70 grams of tomato paste during the same period. The results demonstrated a reduction in oxidative stress and an improvement in endothelial function; the latter is essential to maintain an adequate functioning of the cardiovascular system.

Different scientific studies have suggested that lycopene, as main bioactive compound in the tomato, can exert different beneficial physiological effects for improvements in cardiovascular health *via* platelet aggregation and related vascular mechanisms. For example, Hsiao et al. (38) systematically examined the effects of lycopene in the prevention of platelet aggregation and thrombus formation and proposed two mechanisms of action through the inhibition of the activation of the enzyme phospholipase C and the synthesis of cyclic guanosine monophosphate (GMP-c). Fuentes et al. (39) verified *in vitro* that tomato product intake with a higher concentration of lycopene increased the inhibition of platelet activity induced by various aggregating agents such as adenosine diphosphate (ADP), collagen, arachidonic acid, and the thrombin receptor activator peptide-6 (TRAP-6).

Several studies additionally support an association between plasma and tissue levels of lycopene and both pre-clinical and clinical cardiovascular outcomes. Kong et al. (40) also suggested a beneficial effect of lycopene in the early stages of development and progression of atherosclerosis, as well as in the thickness of the intima-media layer of the carotid artery, a parameter that allows quantifying the level of arterial thickening in preclinical phases of cardiovascular disease. Müller et al. (41) highlighted the powerful antioxidant activity of lycopene that could protect endothelial cells against oxidative stress and prevent the formation of foam cells in the early development of atheroma plaque. Sawardekar et al. (42) indicated that lycopene can exert an antiplatelet effect. Different concentrations of lycopene (4–12 μmol/L) were able to *in vitro* significantly reduce platelet aggregation induced by two aggregating agents, ADP, and collagen. This observed effect was comparable to that exerted by one of the best-known antiplatelet drugs, aspirin. The combination of 4 μmol lycopene/L with 140 μmol aspirin/L showed better results than a single dose of 140 μmol aspirin/L. Phang et al. (43) found an inverse association between plasma and tissue levels of lycopene and the incidence of acute coronary disorders, development of early atherosclerosis, and mortality from heart disease. In addition, Thies et al. (44) showed that subjects with higher lycopene concentrations had a lower risk of suffering a myocardial infarction (59%) and showed a significant improvement in HDL functionality enhancing HDL-antiatherogenic properties.

Clinical trials have also supported the potential beneficial cardiovascular effects attributed to lycopene described above. Klipstein-Grobusch et al. (45), Verghese et al. (46), Kim et al. (47), and Riccioni et al. (48) suggested that lycopene may reduce the risk of atherosclerosis, either directly by attenuating LDL oxidation or indirectly by acting on other cardiovascular risk factors, such as cholesterol. Gajendragadkar et al. (49) conducted a study with 72 individuals, half of them healthy and the other half-undergoing drug treatment because of CVD. There was an improvement in endothelial function in those patients with previous pathologies who ingested a daily amount of 7 mg of lycopene for 2 months. Kim et al. (50) also observed a similar improvement among 37 men with a daily intake of 15 mg of lycopene during the same 8-week follow-up period. These effects for lycopene were attributed to the ability of this bioactive to significantly mitigate oxidative stress and reduce systolic blood pressure.

Other authors have reported a decrease in various cardiovascular risk factors after supplementation with tomato extracts and their derivatives. McEwen (51), Rodríguez-Azúa et al. (52), Palomo et al. (53), Fuentes et al. (39), and Yamamoto et al. (54) suggested that supplementation with tomato extracts may have an antiplatelet effect *in vitro, in vivo* and/or in humans; or even a thrombolytic activity in some tomato variety studied. Both observed activities are very important to avoid the formation of thrombin and, if they have already formed, their dissolution to prevent more serious cardiovascular accidents such as embolisms.

As for WSTC, Uddin et al. (26) carried out a clinical trial with 12 prehypertensive patients who were administered a 150 mg dose of WSTC per day. After 24 h, a significant reduction in blood pressure and platelet aggregation was observed compared to the control group. Likewise, Krasińska et al. (20) indicated a significant hypotensive effect for 213 mg/day of standardized tomato extract administered for 4 weeks in 32 patients with at least high cardiovascular risk based on the European Society of Cardiology (ESC) and the European Society of Hypertension (ESH) in 2013 (55). More recently, O'Kennedy et al. (29) found that daily supplementation with Fruitflow® tomato extract reduces platelet aggregation in humans in response to different cofactors, molecules, and enzymes (ADP, collagen, arachidonic acid, and thrombin) involved in platelet activation. Furthermore, the authors suggested a possible beneficial effect of this tomato extract on some cardiovascular risk factors after intense physical activity that promotes a strong inflammatory response and platelet activation. The effect appeared more pronounced among 6 untrained individuals that had Fruitflow® 90 min before the performance of intense physical activity and significantly reduced markers of inflammation, coagulation, and platelet aggregation compared with controls.

Palomo et al. (25) conducted a pilot study to test whether a tomato pomace extract (by-product) affected platelet aggregation in healthy humans. Tomato pomace extract contains flavonoids as coumaric acid, floridzin, floretin, procyanidin B2, luteolin-7-O-glucoside, kaempferol, and quercetin; as well as nucleosides (adenosine, inosine, and guanosine). The study showed that the daily consumption of 1 g of aqueous extract of tomato pomace for 5 days exerted an inhibitory activity on platelet aggregation.

More recently, investigators have focused their efforts on the role of nutrients and bioactive compounds in helping the immune system to fight against COVID-19 through the diet (56). O'Kennedy and Duttaroy (57) suggest that targeting platelet hyperactivity in the early stages of COVID-19 infection may reduce the immunothrombotic complications of COVID-19 and subdue the systemic inflammatory response. As a result, we believe that the bioactive compounds contained in tomatoes, tomato food products or extracts could meaningfully contribute and promote antioxidant and antiplatelet effects in the human body to complement existing established pharmacologic interventions for the primary and secondary prevention of CVD.

## Conclusions

With regard to the association between tomato products, cardiovascular disease prevention and antiplatelet aggregation, in order to obtain EFSA approval for a related health claim, main research gaps are related to the lack of intervention studies on healthy humans (those with no history of serious disease or hemostatic disorders). At present, with the exception of WSTC studies, most of the clinical trials are performed on individuals with some CV risk factor. With independence of study duration which can be easily fit the 4 weeks required, other difficulty is the use of valid markers to prove the percentage of inhibition in platelet aggregation according to EFSA which should be measured using light transmission aggregometry (LTA) using well-accepted and standardized protocols. Finally, as all components found in the tomato extracts are originally in fresh tomato and tomato products, very convenient and appreciated food products by consumers, future directions on this research topic could be focused on the study of mechanism by which tomatoes and tomato products contribute to cardiovascular health to be considered valued as functional foods.

## Author Contributions

MC, VF-R, and LD conceived the project and design the protocol. MC, VF-R, LD, and RC performed the bibliographic search. MC, VF-R, LD, RC, and M-CS-M performed results analysis. MC and LD wrote the manuscript. HS performed critical review of the manuscript. All authors contributed to the article and approved the submitted version.

## Funding

The authors thank support by UCM ALIMNOVA Research Group (GRFN17/21) and Project OTRI Art. 83 Ref: 317-2020, UCM-Fundación Sabor y Salud. LD is grateful to her PhD grant (UCM-Santander; Ref: CT42/18-CT43/18).

## Conflict of Interest

The authors declare that the research was conducted in the absence of any commercial or financial relationships that could be construed as a potential conflict of interest.

## Publisher's Note

All claims expressed in this article are solely those of the authors and do not necessarily represent those of their affiliated organizations, or those of the publisher, the editors and the reviewers. Any product that may be evaluated in this article, or claim that may be made by its manufacturer, is not guaranteed or endorsed by the publisher.

## References

[1] Cámara M Fernández-Ruiz V Sánchez-Mata MC Domínguez Díaz L Kardinaal A Van Lieshout M. Evidence of antiplatelet aggregation effects from the consumption of tomato products, according to EFSA health claim requirements. Crit Rev Food Sci Nutr. (2019) 60:1515–22. 10.1080/10408398.2019.1577215 30896289

[2] Roth GA Mensah GA Johnson CO Addolorato G Ammirati E Baddour LM. Global burden of cardiovascular diseases and risk factors, 1990–2019. Update from the GBD 2019 study. J Am Coll Cardiol. (2020) 76:2982–3021. 10.1016/j.jacc.2020.11.010 33309175 PMC7755038

[3] Cámara M Sánchez-Mata MC Fernández-Ruiz V Cámara RM Manzoor S Cáceres JO. Lycopene: A Review of Chemical and Biological Activity Related to Beneficial Health Effects en Atta-ur Rahman (Ed.), Studies in Natural Products Chemistry (1st ed., Vol. 40). Amsterdam: Elsevier. (2013) p. 383–426. 10.1016/B978-0-444-59603-1.00011-4

[4] Willcox JK Catignani GL Lazarus S. Tomatoes and cardiovascular health. Crit Rev Food Sci Nutr. (2003) 43:1–18. 10.1080/10408690390826437 12587984

[5] EFSA. Scientific Opinion on the substantiation of health claims related to lycopene and protection of DNA, proteins and lipids from oxidative damage (ID 1608, 1609, 1611, 1662, 1663, 1664, 1899, 1942, 2081, 2082, 2142, 2374), protection of the skin from UV-induced (including photo-oxidative) damage (ID 1259, 1607, 1665, 2143, 2262, 2373), contribution to normal cardiac function (ID 1610, 2372), and maintenance of normal vision (ID 1827) pursuant to Article 13(1) of Regulation (EC) No 1924/2006. (2011). Available online at: https://www.efsa.europa.eu/en/efsajournal/pub/2031. 10.2903/j.efsa.2011.2031 PMC13129911 42078718

[6] European Parliament Council of the European Union. Regulation (EC) No 1924/2006 of the European Parliament and of the Council of 20 December 2006 on nutrition and health claims made on foods. (2006). Available online at: http://eur-lex.europa.eu/legal-content/EN/ALL/?uri¼CELEX%3A02006R1 924-20121129.

[7] EFSA. Scientific Opinion on Lycopene-whey complex (bioavailable lycopene) and risk of atherosclerotic plaques. Scientific substantiation of a health claim related to Lycopene-whey complex (bioavailable lycopene) and reduction of the risk of atherosclerotic plaques pursuant to Article 14 of Regulation (EC) No 1924/2006. (2009) Available online at: https://www.efsa.europa.eu/es/efsajournal/pub/1179

[8] Cámara M Fernández-Ruiz V Fernández Redondo D Sánchez-Mata MC Cámara RM Gervás C. scientific requirements related to lycopene as antioxidant prevention of oxidative damage and cardiovascular health claims. Acta Hortic. (2015) 1081:303–7. 10.17660/ActaHortic.2015.1081.39

[9] Cámara M Fernández-Ruiz V Domínguez L Cámara RM Sánchez-Mata MC. (2018) Lycopene: Regulatory status on its antioxidant health claims en. In: Venketeshwer Rao A Young GL Rao LG editors. Lycopene and Tomatoes in Human Health and Nutrition (1st ed.). London: Taylor & Francis. p. 179–96. 10.1201/9781351110877-10

[10] EFSA. Scientific Opinion on the substantiation of a health claim related to “L-tug lycopene” and reduction of blood LDL-cholesterol pursuant to Article 14 of Regulation (EC) No 1924/2006. (2015). Available online at: https://www.efsa.europa.eu/es/efsajournal/pub/4025.

[11] European Commission. EU Register of nutrition health claims made on food. (2021). Available online at: http://ec.europa.eu/food/safety/labelling_nutrition/claims/register/public/?event=register.home.

[12] O'Kennedy N Raederstorff D Duttaroy AK. Fruitflow®: the first European food safety authority-approved natural cardioprotective functional ingredient. Eur J Nutr. (2017) 56:461–82. 10.1007/s00394-016-1265-2 27388464 PMC5334395

[13] EFSA. Scientific Opinion on water-soluble tomato concentrate (WSTC I and II) and platelet aggregation. Scientific substantiation of a health claim related to water-soluble tomato concentrate (WSTC I and II) and platelet aggregation pursuant to Article 13(5) of Regulation (EC) No 1924/2006. (2009). Available online at: https://www.efsa.europa.eu/en/efsajournal/pub/1101. 10.2903/j.efsa.2009.1101

[14] Cheng HM Koutsidis G Lodge JK Ashor A Siervo M Lara J. Tomato and lycopene supplementation and cardiovascular risk factors: A systematic review and Meta-analysis. Atherosclerosis. (2017) 257:100–8. 10.1016/j.atherosclerosis.2017.01.009 28129549

[15] Cheng HM Koutsidis G Lodge JK Ashor AW Siervo M Lara Lycopene J. and tomato and risk of cardiovascular diseases: a systematic review and meta-analysis of epidemiological evidence. Crit Rev Food Sci Nutr. (2019) 11:1–18. 10.1080/10408398.2017.1362630 28799780

[16] Li N Wu X Zhuang W Xia L Chen Y Wu C. Tomato and lycopene and multiple health outcomes: umbrella review. Food Chem. (2021) 343:128396. 10.1016/j.foodchem.2020.128396 33131949

[17] Rattanavipanon W Nithiphongwarakul C Sirisuwansith P Chaiyasothi T Thakkinstian A Nathisuwan S. Effect of tomato, lycopene and related products on blood pressure: a systematic review and network meta-analysis. Phytomedicine. (2021) 153512. 10.1016/j.phymed.2021.153512 33676812

[18] Tierney AC Rumble CE Billings LM George ES. Effect of Dietary and Supplemental Lycopene on Cardiovascular Risk Factors: a systematic review and meta-analysis. Adv Nutrit. (2020) 11:1453–88. 10.1093/advances/nmaa069 32652029 PMC7666898

[19] Rouhi-Boroujeni H Heidarian E Rouhi-Boroujeni H Deris F Rafieian-Kopaei M. Medicinal plants with multiple effects on cardiovascular diseases: a systematic review. Curr Pharm Des. (2017) 23:999–1015. 10.2174/1381612822666161021160524 27774898

[20] Krasińska B Osińska A Krasińska A Osiński M Rzymski P Tykarski A. Favourable hypotensive effect after standardised tomato extract treatment in hypertensive subjects at high cardiovascular risk: a randomised controlled trial. Kardiol Pol. (2018) 76:388–95. 10.5603/KP.a2017.0215 29131285

[21] Droste DW Iliescu C Vaillant M Gantenbein M De Bremaeker N Lieunard C. Advice on lifestyle changes (diet, red wine and physical activity) does not affect internal carotid and middle cerebral artery blood flow velocity in patients with carotid arteriosclerosis in a randomized controlled trial. Cerebrovascular Dis. (2014) 37:368–75. 10.1159/000362535 24970377

[22] Lazarus SA Bowen K Garg ML. Tomato juice and platelet aggregation in type 2 diabetes. J Am Med Assoc. (2004) 292:805–6. 10.1001/jama.292.7.805 15315994

[23] O'Kennedy N Crosbie L Van Lieshout M Broom JI Webb DJ Duttaroy AK. Effects of antiplatelet components of tomato extract on platelet function *in vitro* and *ex vivo*: a timecourse cannulation study in healthy humans. Am J Clin Nutr. (2006) 84:570–9. 10.1093/ajcn/84.3.570 16960171

[24] O'Kennedy N Crosbie L Whelan S Luther V Horgan G Broom JI. Effects of tomato extract on platelet function: A double-blinded crossover study in healthy humans. Am J Clin Nutr. (2006) 84:561–9. 10.1093/ajcn/84.3.561 16960170

[25] Palomo I Concha-Meyer A Lutz M Said M Sáez B Vásquez A. Chemical characterization and antiplatelet potential of bioactive extract from tomato pomace (Byproduct of Tomato Paste). Nutrients. (2019) 11:456. 10.3390/nu11020456 30813256 PMC6412684

[26] Uddin M Biswas D Ghosh A O'Kennedy N Duttaroy AK. Consumption of fruitflow® lowers blood pressure in prehypertensive males: A randomised, placebo controlled, double blind, cross-over study. Int J Food Sci Nutr. (2018) 69:494–502. 10.1080/09637486.2017.1376621 28918674

[27] Fuentes E Trostchansky A Reguengo LM Junior MRM Palomo I. Antiplatelet effects of bioactive compounds present in tomato pomace. Curr Drug Targets. (2021) 22:1716–24. 10.2174/1389450122999210128180456 33511954

[28] Mozos I Stoian D Caraba A Malainer C Horbanczuk JO Atanasov AG. Lycopene and vascular health. Front Pharmacol. (2018) 9:521. 10.3389/fphar.2018.00521 29875663 PMC5974099

[29] O'Kennedy N Duss R Duttaroy AK. Dietary Antiplatelets: A New Perspective on the Health Benefits of the Water-Soluble Tomato Concentrate Fruitflow®. Nutrients. (2021) 13:2184. 10.3390/nu13072184 34201950 PMC8308204

[30] Olas B. Anti-aggregatory potential of selected vegetables—promising dietary components for the prevention and treatment of cardiovascular disease. Adv Nutrit. (2019) 10:280–290. 10.1093/advances/nmy085 30759176 PMC6416036

[31] Tang G Meng X Li Y Zhao C Liu Q Li H. Effects of vegetables on cardiovascular diseases and related mechanisms. Nutrients. (2017) 9:857. 10.3390/nu9080857 28796173 PMC5579650

[32] Sesso HD Wang L Ridker PM Buring JE. Tomato-based food products are related to clinically modest improvements in selected coronary biomarkers in women. J Nutr. (2012) 142:326–33. 10.3945/jn.111.150631 22223578 PMC3260061

[33] Li YF Chang YY Huang HC Wu YC Yang MD Chao PM. Tomato juice supplementation in young women reduces inflammatory adipokine levels independently of body fat reduction. Nutrition. (2015) 31:691–6. 10.1016/j.nut.2014.11.008 25837214

[34] Hsu YM Lai CH Chang CY Fan CT Chen CT Wu CH. Characterizing the lipid-lowering effects and antioxidant mechanisms of tomato paste. Biosci Biotechnol Biochem. (2008) 72:677–85. 10.1271/bbb.70402 18323670

[35] Burton-Freeman B Talbot J Park E Krishnankutty S Edirisinghe I. Protective activity of processed tomato products on postprandial oxidation and inflammation: A clinical trial in healthy weight men and women. Mol Nutr Food Res. (2012) 56:622–31. 10.1002/mnfr.201100649 22331646

[36] García-Alonso FJ Jorge-Vidal V Ros G Periago MJ. Effect of consumption of tomato juice enriched with n-3 polyunsaturated fatty acids on the lipid profile, antioxidant biomarker status, and cardiovascular disease risk in healthy women. Eur J Nutr. (2012) 51:415–24. 10.1007/s00394-011-0225-0 21755327

[37] Xaplanteris P Vlachopoulos C Pietri P Terentes-Printzios D Kardara D Alexopoulos N. Tomato paste supplementation improves endothelial dynamics and reduces plasma total oxidative status in healthy subjects. Nutrit Res. (2012) 32:390–4. 10.1016/j.nutres.2012.03.011 22652379

[38] Hsiao G Wang Y Tzu NH Fong TH Shen MY Lin KH. Inhibitory effects of lycopene on *in vitro* platelet activation and *in vivo* prevention of thrombus formation. J Lab CliniMed. (2005) 146:216–26. 10.1016/j.lab.2005.03.018 16194683

[39] Fuentes E Carle R Astudillo L Guzman L Gutierrez M Carrasco G. Antioxidant and antiplatelet activities in extracts from green and fully ripe tomato fruits (*Solanum lycopersicum*) and pomace from industrial tomato processing. Evid Based Complementary Altern. (2013) 867578. 10.1155/2013/867578 23476707 PMC3588208

[40] Kong KW Khoo HE Prasad KN Ismail A Tan CP Rajab NF. Revealing the power of the natural red pigment lycopene. Molecules. (2010) 15:959–87. 10.3390/molecules15020959 20335956 PMC6263198

[41] Müller L Caris-Veyrat C Lowe G Böhm V. Lycopene and its antioxidant role in the prevention of cardiovascular diseases—a critical review. Crit Rev Food Sci Nutr. (2016) 56:1868–79. 10.1080/10408398.2013.801827 25675359

[42] Sawardekar SB Patel TC Uchil D. Comparative evaluation of antiplatelet effect of lycopene with aspirin and the effect of their combination on platelet aggregation: an *in vitro* study. Indian J Pharmacol. (2016) 48:26–31. 10.4103/0253-7613.174428 26997718 PMC4778201

[43] Phang M Lazarus S Wood LG Garg M. Diet and thrombosis risk: nutrients for prevention of thrombotic disease. Semin Thromb Hemost. (2011) 37:199–208. 10.1055/s-0031-1273084 21455854

[44] Thies F Mills LM Moir S Masson LF. Cardiovascular benefits of lycopene: fantasy or reality? Proc Nutr Soc. (2017) 76:122–9. 10.1017/S0029665116000744 27609297

[45] Klipstein-Grobusch K Launer LJ Geleijnse JM Boeing H Hofman A Witteman JC. Serum carotenoids and atherosclerosis. The Rotterdam study. Atherosclerosis. (2000) 148:49–56. 10.1016/S0021-9150(99)00221-X 10580170

[46] Verghese M Richardson JE Boateng J Shackelford LA Howard C Walker LT. Dietary lycopene has a protective effect on cardiovascular disease in New Zealand male rabbits. J Biological Sci. (2008) 8:268–77. 10.3923/jbs.2008.268.277

[47] Kim OY Yoe HY Kim HJ Park JY Kim JY Lee SH. Independent inverse relationship between serum lycopene concentration and arterial stiffness. Atherosclerosis. (2010) 208:581–6. 10.1016/j.atherosclerosis.2009.08.009 19767001

[48] Riccioni G Scotti L Di Ilio E Bucciarelli V Ballone E De Girolamo M. Lycopene and preclinical carotid atherosclerosis. J Biol Regul Homeost Agents. (2011) 25:435–41. Available online at: https://www.researchgate.net/publication/51739032_Lycopene_and_preclinical_carotid_atherosclerosis 22023768

[49] Gajendragadkar PR Hubsch A. Mäki-Petäjä KM Serg M Wilkinson IB Cheriyan J. *Effects of oral lycopene supplementation on vascular function in patients with* Cardiovascular disease and healthy volunteers: a randomised controlled trial. PLoS ONE. (2014) 9:e99070. 10.1371/journal.pone.0099070 24911964 PMC4049604

[50] Kim JY Paik JK Kim OY Park HW Lee JH Jang Y. Effects of lycopene supplementation on oxidative stress and markers of endothelial function in healthy men. Atherosclerosis. (2011) 215:189–95. 10.1016/j.atherosclerosis.2010.11.036 21194693

[51] McEwen BJ. The influence of diet and nutrients on platelet function. Semin Thromb Hemost. (2014) 40:214–26. 10.1055/s-0034-1365839 24497119

[52] Rodríguez-Azúa R Treuer A Moore-Carrasco R Cortacans D Gutiérrez M Astudillo L. Effect of tomato industrial processing (different hybrids, paste, and pomace) on inhibition of platelet function *in vitro, ex vivo, in vivo*. J Med Food. (2014) 17:505–11. 10.1089/jmf.2012.0243 24325459 PMC3993053

[53] Palomo I Fuentes E Padro T Badimon L. Platelets and atherogenesis: platelet antiaggregation activity and endothelial protection from tomatoes (*Solanum lycopersicum* L.). Exp Ther Med. (2012) 23:109–11. 10.3892/etm.2012.477 22969932 PMC3438755

[54] Yamamoto J Taka TK Yamada Y Ijiri M Murakami Y Hirata A. Tomatoes have natural anti-thrombotic effects. Br J Nutrit. (2003) 90:1031–8. 10.1079/BJN2003988 14641962

[55] Mancia G Fagard R Narkiewicz K Redon J Zanchetti A Böhm M. 2013 ESH/ESC Guidelines for the management of arterial hypertension: the task force for the management of arterial hypertension of the European Society of Hypertension (ESH) and of the European Society of Cardiology (ESC). Eur Heart J. (2013) 34:2159–219. 10.1093/eurheartj/eht151 23771844

[56] Cámara M Sánchez-Mata MC Fernández-Ruiz V Cámara RM Cebadera E Domínguez L. Review of the role of micronutrients and bioactive compounds on immune system supporting to fight against the COVID-19 disease. Foods. (2021) 10:1088. 10.3390/foods10051088 34068930 PMC8155867

[57] O'Kennedy N Duttaroy AK. Platelet hyperactivity in COVID-19: can the tomato extract fruitflow® be used as an antiplatelet regime? Med Hypotheses. (2021) 147:110480. 10.1016/j.mehy.2020.110480 33421690 PMC7781513

