# Supplementary figures and images for: Scientific Evidence of the Beneficial Effects of Tomato Products on Cardiovascular Disease and Platelet Aggregation
Source: Front Nutr. 2022 Mar 15;9:849841. doi: 10.3389/fnut.2022.849841 (PMC8965467; doi:10.3389/fnut.2022.849841)

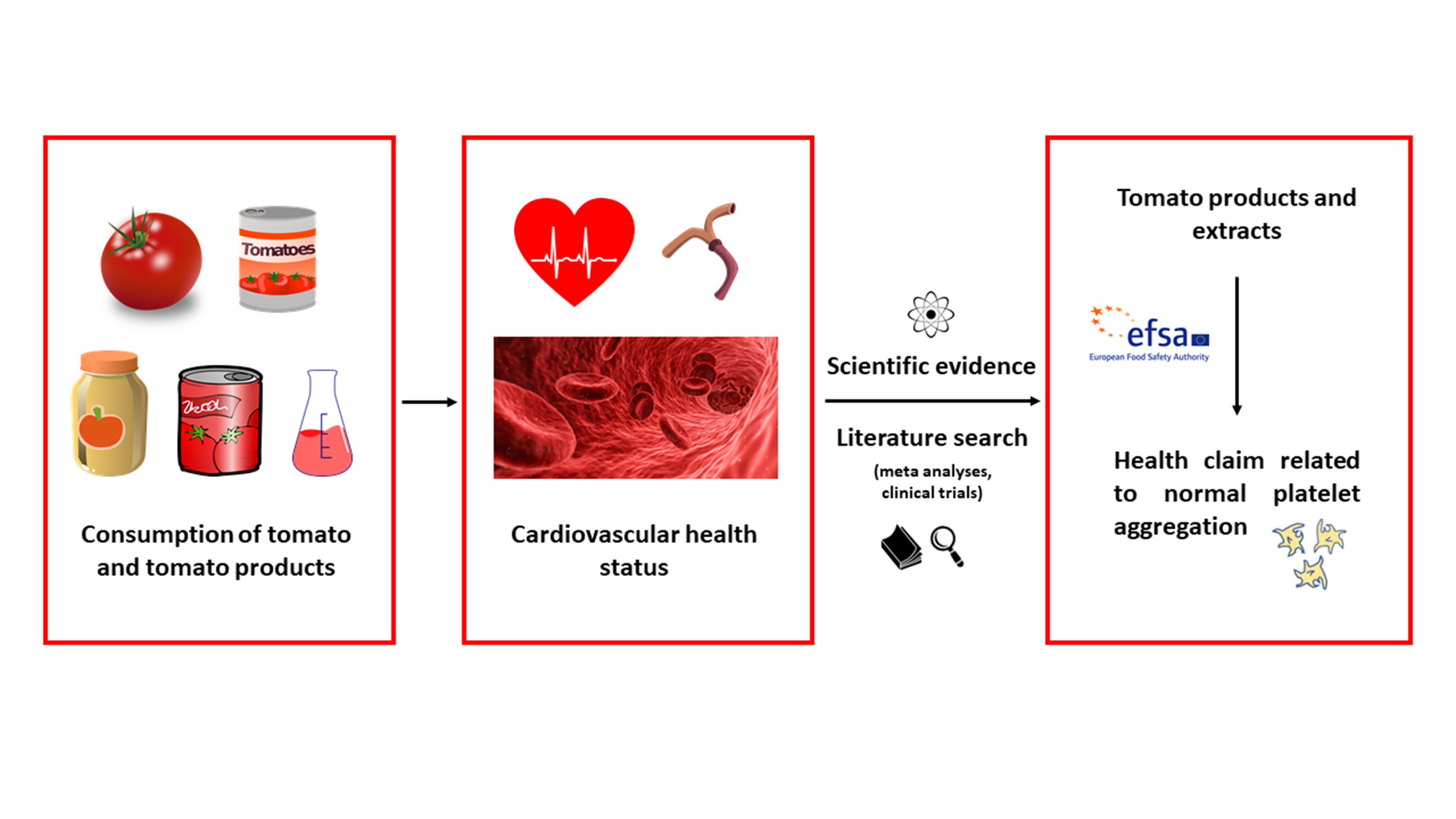

Supplement: Supplementary file 1 [file Image_1.tif]
